# Structure of the SthK Carboxy-Terminal Region Reveals a Gating Mechanism for Cyclic Nucleotide-Modulated Ion Channels

**DOI:** 10.1371/journal.pone.0116369

**Published:** 2015-01-27

**Authors:** Divya Kesters, Marijke Brams, Mieke Nys, Eveline Wijckmans, Radovan Spurny, Thomas Voets, Jan Tytgat, Jana Kusch, Chris Ulens

**Affiliations:** 1 Laboratory of Structural Neurobiology, KU Leuven, Herestraat 49, PB601, Leuven, B-3000, Belgium; 2 Laboratory of Ion Channel Research, KU Leuven, Herestraat 49, PB802, Leuven, B-3000, Belgium; 3 Laboratory of Toxicology and Pharmacology, KU Leuven, Herestraat 49, PB922, Leuven, B-3000, Belgium; 4 University Hospital Jena, Institute of Physiologie II, Kollegiengasse 9, 07743, Jena, Germany; Zhejiang University, CHINA

## Abstract

Cyclic nucleotide-sensitive ion channels are molecular pores that open in response to cAMP or cGMP, which are universal second messengers. Binding of a cyclic nucleotide to the carboxyterminal cyclic nucleotide binding domain (CNBD) of these channels is thought to cause a conformational change that promotes channel opening. The C-linker domain, which connects the channel pore to this CNBD, plays an important role in coupling ligand binding to channel opening. Current structural insight into this mechanism mainly derives from X-ray crystal structures of the C-linker/CNBD from hyperpolarization-activated cyclic nucleotide-modulated (HCN) channels. However, these structures reveal little to no conformational changes upon comparison of the ligand-bound and unbound form. In this study, we take advantage of a recently identified prokaryote ion channel, SthK, which has functional properties that strongly resemble cyclic nucleotide-gated (CNG) channels and is activated by cAMP, but not by cGMP. We determined X-ray crystal structures of the C-linker/CNBD of SthK in the presence of cAMP or cGMP. We observe that the structure in complex with cGMP, which is an antagonist, is similar to previously determined HCN channel structures. In contrast, the structure in complex with cAMP, which is an agonist, is in a more open conformation. We observe that the CNBD makes an outward swinging movement, which is accompanied by an opening of the C-linker. This conformation mirrors the open gate structures of the K_v_1.2 channel or MthK channel, which suggests that the cAMP-bound C-linker/CNBD from SthK represents an activated conformation. These results provide a structural framework for better understanding cyclic nucleotide modulation of ion channels, including HCN and CNG channels.

## INTRODUCTION

Hyperpolarization-activated cyclic nucleotide-modulated (HCN) channels and cyclic nucleotide-gated (CNG) channels belong to the superfamily of six-transmembrane domain K^+^ channels (for reviews, see references [1–4]). HCN channels play an important role in generating spontaneous rhythmic firing of action potentials in excitable cells and are known targets for a class of antiarrhythmic agents, including ivabradine [5, 6]. Additionally, mutations in HCN channel genes are responsible for inherited disorders, such as sinus bradycardia [7]. CNG channels are important for signal transduction in retinal photoreceptors and olfactory sensory neurons [3]. Inherited mutations in CNG channel genes are linked to total color blindness [8].

At the molecular level, both HCN and CNG channels have a similar topology and are composed of six transmembrane domains (S1–S6) containing a putative voltage sensor (S4) and a pore-forming domain (S5–S6), which connects to a cytoplasmic C-linker region and a cyclic nucleotide-binding domain (CNBD). CNG channels are solely gated by cyclic nucleotides, cAMP or cGMP, whereas HCN channels are gated by voltage and modulated by cyclic nucleotides. Binding of cyclic nucleotides to the CNBD is thought to cause a conformational change in the C-linker/CNBD, which opens the channel gate and allows the flux of ions through the channel pore. The C-linker region, which connects the CNBD to the channel pore, critically contributes to coupling of ligand binding to channel opening (reviewed in reference [3]).

Current structural insight into the mechanism of channel modulation and ligand specificity derives from crystal structures of the carboxyterminal C-linker/CNBD of mammalian or invertebrate HCN channels [9–12]. These structures reveal a tetrameric assembly mirroring the symmetry of the tetrameric channel pore. The tetramer interface of the C-linker/CNBD region is mainly formed by the C-linker, which is composed of six α-helices, termed αA’ to αF’. At the subunit interface, the A’-B’ helices from one subunit form an ‘elbow’ resting on the ‘shoulder’ (C’-D’ helices) from a neighboring subunit. Furthermore, previous C-linker/CNBD structures have shown that the CNBD is composed of a so-called β-roll flanked by two α-helices (A and B) and a carboxyterminal C-helix, which directly interacts with the ligand. In addition, crystal structures have also been determined for the C-linker and cyclic nucleotide binding homology domain (CNBHD) of related ion channels, including the zebrafish EAG-like (ELK) K^+^ channel [13], the mosquito ERG K^+^ channel [14], and the mouse EAG1 K^+^ channel [15]. Despite this wealth of structural information, insight into the molecular architecture of the carboxyterminal region of CNG channels is still lacking. Moreover, all of the HCN channel crystal structures of the C-linker/CNBD regions determined to date reveal a nearly identical architecture in the absence and presence of ligand [16], which raises an important question what the conformational changes are in this region that underlie cyclic nucleotide modulation of HCN and CNG channels.

In this study, we take advantage of a recently identified prokaryote homologue of HCN and CNG channels, termed SthK, which originates from the thermophylic bacterium *Spirochaeta thermophila* [17]. We previously demonstrated that SthK is gated by cAMP, but not cGMP, and is relatively insensitive to voltage, which resembles the gating properties of certain eukaryote CNG channels (see S1 Fig.). Using X-ray crystallography, we determined the 3-dimensional structures of the isolated C-linker/CNBD of SthK, termed SthK-C_term_, in complex with cAMP or cGMP. We observe that the SthK-C_term_ structure in complex with cGMP strongly resembles previously published HCN channel structures. In contrast, in the SthK-C_term_ structure in complex with cAMP important differences exist. Here, the C-linker adopts a conformation in which the A’-helix, which connects to the gate of the channel, is bent and in a more open conformation compared to the HCN structures. In addition, we observe that the CNBD undergoes an outward (i.e. away from the pore axis) swinging motion, which is accompanied by a movement of the B- and C-helices. The cAMP-bound SthK-C_term_ structure mirrors the open gate structure of the K_v_1.2 channel [18] and MthK channel [19] and suggests that this C-linker/CNBD possibly represents an activated conformation. Our results contribute to a better understanding of the conformational changes that underlie cyclic nucleotide modulation in HCN and CNG channels.

## EXPERIMENTAL PROCEDURES

### Protein expression and purification

The DNA fragment encoding amino acid residues 226–430 of the full length sequence was cloned as a synthetic gene into the *Nco*I and *Hind*III restriction sites of pHMalc2T [12], which is a derivative of pMalc2T (New England Biolabs) containing a hexahistidine tag at the N-terminus of maltose-binding protein (MBP). This construct was transformed into *Escherichia coli* BL21(DE3) cells and a cell culture was grown at 37°C in Luria broth to an OD_600 nm_ of 0.6–0.8. Protein expression was induced by addition of 0.4 mM IPTG and the culture was incubated at 18°C overnight. Cells from a 1 L culture were harvested by centrifugation at 10,000 g and resuspended in 50 mL buffer A, containing 150 mM NaCl, 30 mM HEPES pH 8.0, 5 mM cAMP, 1 mM DDT and 10% glycerol, supplemented with 1 mM PMSF, 20 mg/mL DNase, 5 mM MgCl_2_, 1 mg/mL leupeptin, 1 mg/mL pepstatin. Cells were lysed by 2 passes through an Emulsiflex-C5 high pressure homogenizer (Avestin). The cell lysate was cleared by centrifugation at 27,000 g for 15 min. The clear supernatant was incubated with 3 mL pre-washed amylose resin (New England Biolabs) for 1–2 hour at 4°C. The resin was collected on a column and washed with 15 column volumes of buffer A. Bound protein was eluted in 5 column volumes buffer A supplemented with 50 mM maltose. Protein concentration was measured by absorbance at 280 nm using a Nanodrop 2000 spectrophotometer (Thermo Scientific). Protein was diluted to a concentration of 3.5 mg/ml and MBP-fusion protein was cleaved with 500 units of thrombin (Calbiochem) overnight at 4°C. Protein was concentrated on a Amicon ultra filter device with a molecular weight cut-off of 50,000 Da (Millipore) and loaded on a HiLoad Superdex 200 gel filtration column (GE Healthcare) pre-equilibrated with buffer containing 30 mM HEPES pH 8.0, 500 mM NaCl, 1 mM DTT and 10% glycerol. The tetrameric stoichiometry of SthK-C_term_ was confirmed with a multi-angle laser light system (MALLS) coupled to size exclusion chromatography (shown as S2 Fig.). Fractions corresponding to SthK-C_term_ were pooled and incubated with 1 mL amylose resin for 1 h. Protein was concentrated to 10 mg/mL, snap frozen in liquid nitrogen and stored at -80°C.

### Crystallography

Crystals were grown at 20°C using the sitting-drop vapor diffusion method. cAMP or cGMP were added to the protein at a final concentration of 3 mM or 5 mM cGMP, respectively. Crystallization screens were set up by mixing 100 nL of the concentrated protein with reservoir solutions at a 1:1 ratio by a Mosquito nanoliter crystallization robot (TTP Labtech). Crystal growth was monitored using a RockImager (Formulatrix) and for SthK-C_term_ in complex with cAMP a crystallization hit was obtained in the following condition: 100 mM MMT buffer system (DL-malic acid, 2-(N-morpholino)ethanesulfonic acid, Tris 1:2:2) pH 5.0 and 25% (w/v) PEG1500. For SthK-C_term_ in complex with cGMP a crystallization hit was obtained in the following condition: 200 mM disodium-malonate, 100 mM bistrispropane pH 7.5 and 20% (w/v) PEG3350. Crystals were harvested in mother liquor and cryo-protected by the addition of 30% glycerol in 5% increments. Crystals were flash-cooled by immersion in liquid nitrogen. From more than 100 crystals tested, a few diffracted X-rays to a resolution better than 2.5Å and diffraction data sets were collected at the X06A beam line of the Swiss Light Source (SLS, Villigen) and the ID23–2 beam line of the European Synchrotron Radiation Facility (ESRF, Grenoble). The structure of SthK-C_term_ in complex with cAMP was determined by molecular replacement with MOLREP using a crystallographic tetramer of the HCN4 carboxyterminal domain (pdb code 3u11) as a search model, which was first pruned using Chainsaw [20]. The asymmetric unit contained 2 C-linker/CNBD subunits, termed chain A and chain B. The electron density map for chain A was of sufficiently high quality to permit building of residues 226 to 423. However, a small number of side chains in chain A were not built due to poor electron density. The electron density map for chain B was of notably lower quality and therefore only chain A has been used for the structure analysis. Initial structure refinement was done with Refmac [20] and an improved model was obtained using Autobuild in PHENIX [21]. The structure was further improved using iterative cycles of manual rebuilding in Coot and automated refinement in PHENIX [21]. The structure of SthK-C_term_ in complex with cGMP was solved by molecular replacement with the cAMP-bound SthK-C_term_ from which the ligand was removed. Structure validation was done with Molprobity [22] and figures were prepared in Pymol (Schrödinger).

## RESULTS

**Fig 1:**
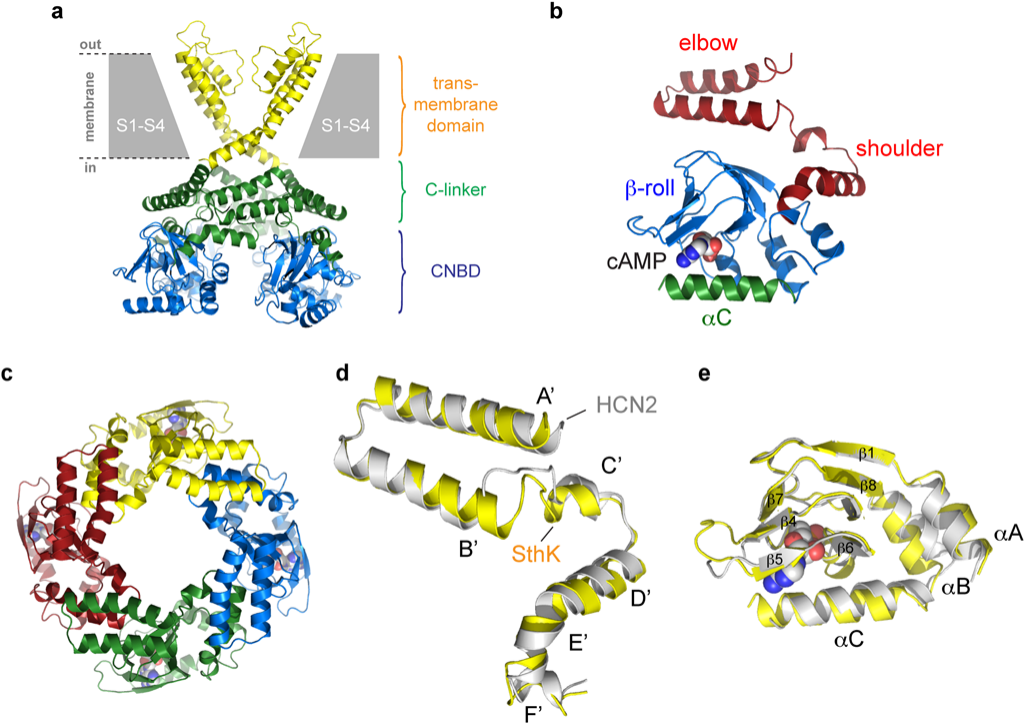
Molecular architecture of the SthK C-terminal domain. **a)** Superposition of the SthK-C_term_ tetramer onto the open gate structure of the Kv1.2 channel. The CNBD is shown in blue, the C-linker in green and the transmembrane domain in yellow. Only two transmembrane subunits are shown for clarity. **b)** Cartoon representation of a single SthK-C_term_ monomer. The C-linker is shown in red, the β-roll in blue and the C-helix in green. The cAMP molecule is shown in sphere representation. Blue atoms are nitrogen, white atoms are carbon and red atoms are oxygen. **c)** Top down cartoon view of the SthK-C_term_ tetramer along the fourfold symmetry axis. Each of the four subunits is shown in a different color. **d)** Superposition of the C-linker of SthK (yellow) onto HCN2 (white). **e)** Superposition of the CNBD of SthK (yellow) onto HCN2 (white)

The structures of the SthK C-terminal domain, termed SthK-C_term_, were determined in complex with cAMP or cGMP at 2.6Å resolution. Crystallographic statistics are reported in detail in Table 1 and crystal packing for the two complexes is shown in S3 Fig. The electron density map was of sufficiently high quality to permit building of residues 226 to 423. The crystal structure of SthK-C_term_ in complex with cGMP, which is an antagonist, adopts an overall architectural fold that strongly resembles that of previous structures of HCN C-terminal domains. The root mean square deviation (r.m.s.d.) for the α-carbons of SthK-C_term_ and mouse HCN2 in complex with cGMP (pdb entry 1q3e) is 1.5Å for 184 α-carbons superposed. In contrast, the structure of SthK-C_term_ in complex with cAMP, which is an agonist, displays significant differences (Fig. 1a-c). Similar to HCN channels, the structure of cAMP-bound SthK-C_term_ reveals a C-linker domain composed of six α-helices (αA’ to αF’), with the A’- and B’-helices forming an antiparallel helix-turn-helix motif (Fig. 1d). The C-linker domain is followed by the CNBD, which contains four helices (αA, αB, αP, αC) and eight β-strands arranged in a so-called β-roll, which is flanked by the A- and B-helices (Fig. 1e). Although the overall fold of the carboxyterminal domain of SthK and HCN channels are relatively similar, superposition of the cAMP-bound structures of SthK and mouse HCN2 reveal significant differences. The r.m.s.d. for the α-carbons of the two structures is 2.1Å (182 α-carbons superposed), 1.8Å for the C-linker domain superposed alone (78 α-carbons) and 1.3Å for the CNBD superposed alone (111 α-carbons). For the C-linker domain, the most notable differences are in the N-terminal half of the A’-helix (Fig. 1d), which connects to the S6 segment of the pore domain, and tilts out- and upward by a distance of 4Å compared to HCN2. Smaller differences can be seen in the C’-helix and D’-E’-F’-helices which undergo an outward shift (i.e. away from the pore axis) by less than 2Å. For the CNBD, the β-sheet core of the β-roll is very similar, but most of the differences can be seen in the B-helix, which tilts outward by a distance of 3–4Å (Fig. 1e).

**Table 1:** 

	**SthK-C_term_ in complex with cAMP**	**SthK-C_term_ in complex** **with cGMP**
Crystallographic statistics		
Beamline	X06A (SLS)	ID23–2 (ESRF)
Date of collection	30-Mar-14	20-Jun-2014
Wavelength (Å)	1.0000	0.872600
Spacegroup	*P*4	*I*4
*a, b, c* (Å)	87.439, 87.439, 79.468	87.202 87.202 142.337
*α, β, γ* (°)	90.00, 90.00 90.00	90.00 90.00 90.00
Resolution limits (Å)	48.80–2.58 (2.72–2.58)	46.61–2.55 (2.68–2.55)
*R_merge_* (%)	4.7 (75.0)	6.8 (71.5)
*<I/σ>*	15.1 (1.6)	10.6 (1.5)
Multiplicity	3.4 (3.1)	3.2 (3.1)
Completeness (%)	99.1 (96.6)	99.7 (99.9)
Total number of reflections	63119 (8153)	54301 (7759)
Number unique reflections	18751 (2635)	17256 (2522)
Refinement and model statistics		
R_work_ (%)	26.0	19.8
R_free_ (%)	30.0	25.4
Rmsd bond distance (Å)	0.0034	0.002
Rmsd bond angle (°)	0.761	0.704
Ramachandran analysis		
Outliers (%)	0	0.3
Favored (%)	92.0	96.1
Average B-factors		
Chain A	91.1	74.4
Chain B	121.3	80.2
Ligand	86.5	62.4
Water	77.2	72.0

**Fig 2:**
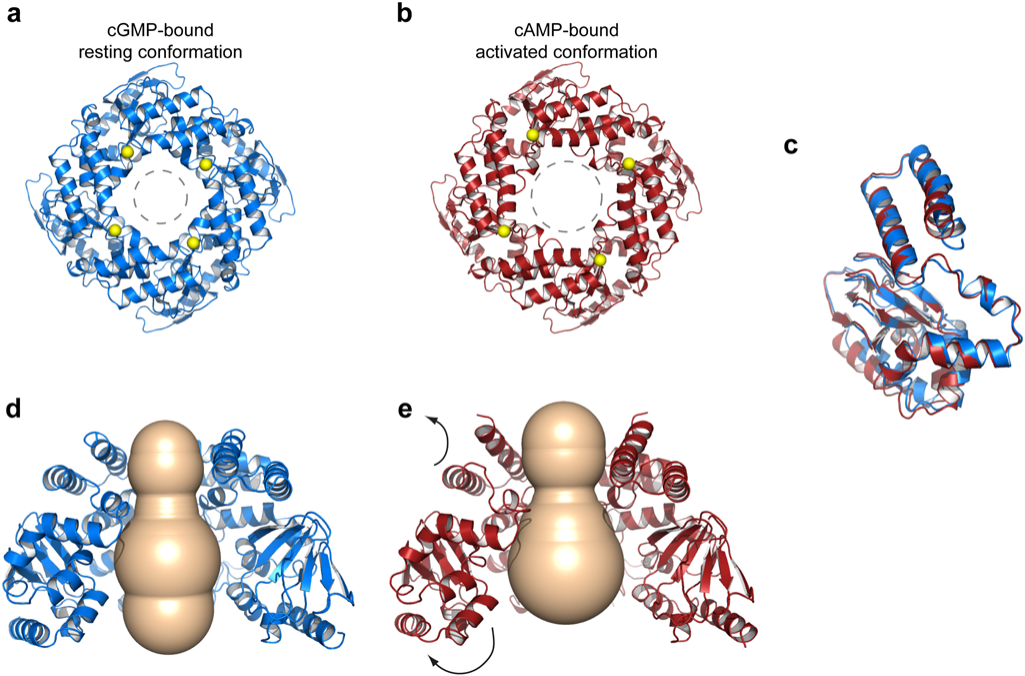
The SthK-C_term_ structure in complex with the agonist cAMP is in a more open conformation than in the complex with the antagonist cGMP. **a)** Top down cartoon view of the SthK-C_term_ structure in complex with cGMP is shown in blue **b)** Same view as in a) for SthK-C_term_ in complex with cAMP, shown in red. The yellow spheres indicate the position where the C-linker starts and is also the site where the C-linker connects to the gate of the channel. **c)** Differences in monomer conformation after superposition of the cAMP-bound tetramer onto the cGMP-bound tetramer. Blue is cGMP-bound, red is cAMP-bound. **d)** Side view of SthK-C_term_ in complex with cGMP with one subunit omitted for clarity. **e)** same view as in d) but for SthK-C_term_ in complex with cAMP.

The SthK-C_term_ subunits form a tetramer that radially assembles along a four-fold symmetry axis that extends to the ion channel pore of the transmembrane domain. The tetramer interface of SthK-C_term_ is mainly formed by the C-linker domain, with the ‘elbow’ of one subunit resting on the ‘shoulder’ of its neighboring subunit (Fig. 1a-c). Superposition of the cAMP-bound SthK tetramer onto the cGMP-bound SthK tetramer reveals an important change in the quaternary structure (Fig. 2). The r.m.s.d. for the two structures is 1.8Å for 772 α-carbons superposed, with a clear conformational change of the C-linker domain relative to the CNBD (Fig. 2a-c). The CNBD makes an outward swinging movement, which is most obvious in the P-, B-, and C-helices. This causes a significant widening of the SthK tetramer pore, which is most pronounced near the CNBD (Fig. 2d-e). In the C-linker domain, the D’-E’-F’-helices remain relatively constant, whereas the C’-helix makes an outward motion and the A’-helix undergoes an upward (i.e. toward the membrane side) and outward bending. The distance measured between the α-carbons of neighboring aminoterminal residues amounts 28.6Å in cAMP-bound SthK compared to 23.8Å in cGMP-bound SthK (indicated as yellow spheres in Fig. 2a-b). The bigger distance in cAMP-bound SthK mirrors the distance measured at the carboxyterminal end of the open gate in the structure of the Kv1.2 channel (29.7 Å) and the MthK channel (30.3Å). This observation suggests that the cAMP-bound structure of SthK-C_term_ represents an activated conformation. In line with recent double electron-electron resonance data [23], the superposition of cAMP-bound and cGMP-bound SthK-C_term_ shows that there are no conformational differences in the β-roll region from the CNBD upon cAMP binding. However, significant conformational changes are present in the αP-, αB- and αC-helix, which make a clear outward movement compared to the cGMP-bound structure (Fig. 2d-e). A smaller shift can be seen for the αA, αF’ and αE’. The conformational change of SthK-C_term_ upon binding of cAMP is further illustrated with a morphing movie, which is available as supplementary material.

**Fig 3:**
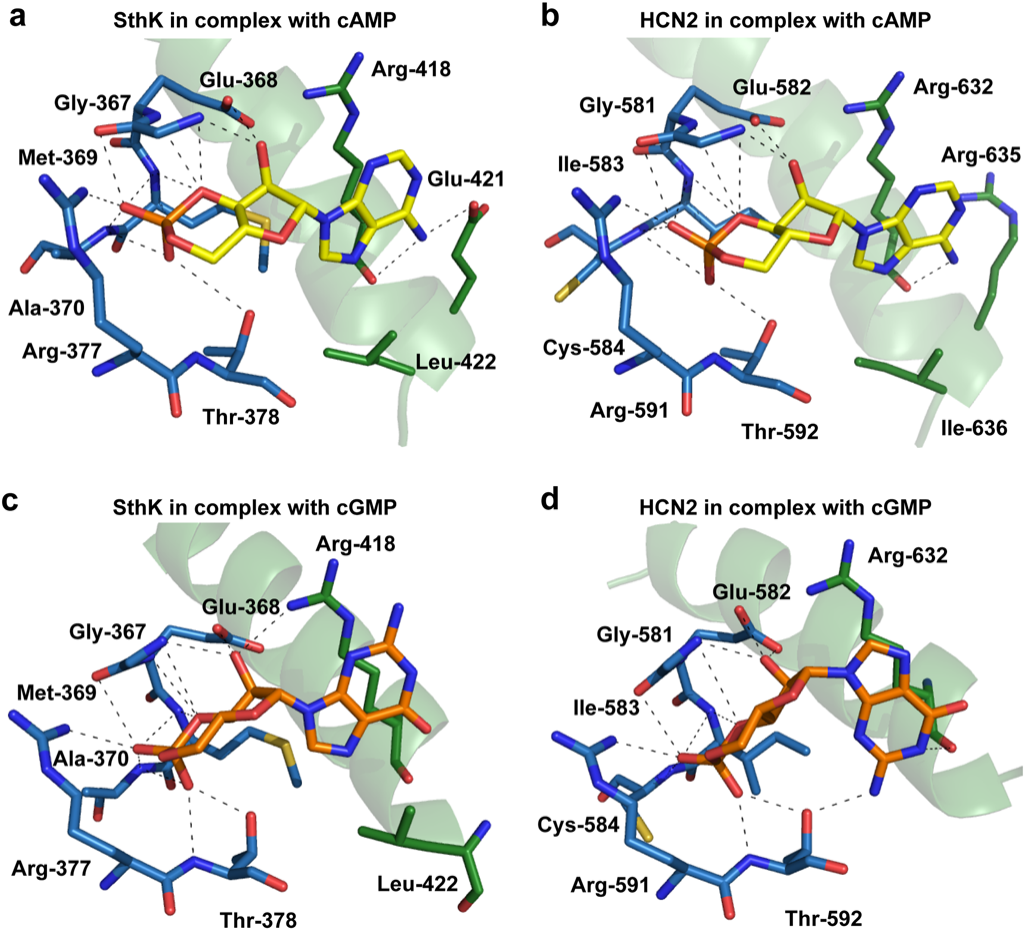
Molecular determinants of ligand recognition in the SthK-C_term_ in complex with cAMP or cGMP. **a-b)** Comparison of the amino acids in the ligand binding site involved in recognition of cAMP in SthK (left) and HCN2 (right). Amino acids of the β-roll are shown in blue sticks, residues of the C-helix in green sticks. The C-helix is shown in cartoon representation. The cAMP molecule is shown in yellow. Dashed lines represent hydrogen bonds or salt bridges. **c-d)** Comparison of the cGMP bound SthK-C_term_ with the cGMP bound structure of HCN2 illustrates that the cGMP molecule binds in the anti-conformation, whereas previously published structures showed cGMP bound in its syn-conformation. Locked in the anti-conformation, the phosphoribose part of cGMP is able to establish the same interaction with SthK-CNBD as cAMP. However, the anti-conformation appears to inhibit interactions between the purine ring from cGMP and the C-helix from the SthK-CNBD. Most likely, the absence of these interactions explains why binding of cGMP to SthK does not provide the required energy for the opening conformational changes.

Next, we investigated whether the molecular determinants for cAMP and cGMP recognition are conserved between SthK and eukaryote HCN channels. Similar to HCN channels, the cAMP molecule is located in a binding cavity that is formed by the β-roll and C-helix from the CNBD of SthK-C_term_ (Fig. 3a-b). The phosphoribose group from cAMP is buried into this binding cavity whereas the adenosine ring is pointing towards the opening of the binding cavity. Similar as in the previously shown mouse HCN2 and sea urchin SPIH structures, the cAMP molecule is bound in an anti-conformation. Besides the overall similar orientation of cAMP in the CNBD from SthK-C_term_, the individual protein-ligand interactions also seem to be very well conserved between the prokaryote SthK CNBD and the eukaryote CNBD from HCN2 and SPIH. The interaction blueprint around the phosphoribose group from cAMP with SthK-C_term_ looks almost identical to the one shown for HCN2 (Fig. 3a-b). The phosphoribose from cAMP interacts with residues Gly-367, Glu-368, Arg-377 and Thr-378 (resp. Gly-581, Glu-582, Arg-591 and Thr-592 in HCN2 and Gly-610, Glu-611, Arg-620 and Val-621 in SPIH). In HCN2, Ile-583 and Cys-584 are also lining the binding cavity around the phosphoribose group but these residues are not conserved in the SthK CNBD (Met-369 and Ala-370, resp.). However, protein-ligand interactions at this location appear to be established mainly by backbone interactions and therefore Met-369 and Ala-370 in SthK are able to accomplish the same ligand interactions. Furthermore, protein-ligand interactions are also conserved around the purine ring from cAMP. Arg-632 from HCN2, which has been shown to play an important role in ligand binding through interaction with N6 via a hydrogen bound, is clearly conserved in the SthK CNBD (Arg-418). In that same region we could also see that other key residues such as Val-348 and Leu-361 (Val-564 & Leu-574 in HCN2 and Ile-592 & Leu-603 in SPIH) are also conserved in the SthK CNBD. Additionally, it is known that HCN2 and SPIH line the binding cavity near the purine ring with polar residues, Met-572 and Thr-601, respectively. The CNBD from SthK has an alanine (Ala-358) located at this position. However, lack of interaction between this Ala-358 and N6 from cAMP appears to be compensated by the presence of Glu-421 in SthK-C_term_, which forms a strong hydrogen bound with N6. All together, analysis of the interactions in the binding pocket shows that the cAMP molecule binds in a similar way as shown for HCN2 and SPIH. This result confirms that the molecular determinants for ligand recognition are highly conserved in SthK-C_term_, despite the distant evolutionary relationship between SthK and eukaryote HCN channels. In the structure of cGMP-bound SthK-C_term_ we observe that cGMP is able to bind in the cyclic nucleotide binding cavity from SthK (Fig. 3c). Moreover, the phosphoribose group from cGMP establishes the same interactions that were described earlier for the phosphoribose group from cAMP in the SthK-C_term_ structure. Remarkably, we observe that cGMP is bound in its anti-conformation in SthK-C_term_ whereas previously published cGMP-bound CNBD structures showed the cGMP molecule bound in its syn conformation (Fig. 3d) [9, 12]. The anti-conformation in the cGMP-bound SthK-C_term_ structure prevents the purine ring of cGMP from establishing binding interactions with the C-helix or Thr-378 from the CNBD (Fig. 3c-d) [12]. Therefore, it is possible that antagonism by cGMP arises from missing interactions between the purine ring of cGMP and the C-helix of SthK.

## DISCUSSION

Our results support a molecular mechanism in which agonist binding to the C-linker/CNBD results in channel modulation via allosteric conformational changes which involve movement of the C-helix relative to the β-roll together with a global upward and outward movement of the C-linker/CNBD. These results thereby extend on previous insight derived from studies investigating the dynamic properties of the C-linker/CNBD using various techniques. First, disulfide cross-linking studies between introduced cysteines in the C-helix demonstrated that disulfide bridge formation primarily occurred in closed channels and inhibited channel activation. This result indicated that the C-helices are in close proximity in the closed state and move apart during channel opening [24, 25]. Furthermore, the role of C-helix movement towards the β-roll has been proposed based on various mutagenesis studies on the CNGA1 channel. One of these studies showed that substitution of residue D604 in the CNGA1 C-helix has major effects on the allosteric transitions of the C-linker-CNBD [26]. The role of the C-helix movement was further demonstrated using the substituted cysteine accessibility method (SCAM) in which modulation of a specific residue in the C-helix of CNGA1, G597C, prevented the allosteric transitions that are required for channel opening even though the G597C modulation did not appear to interfere with initial ligand binding to the C-linker/CNBD [27]. Based on mutagenesis studies by Flynn *et. al* (2007) on the cyclic nucleotide modulated channel SpIH, a modulation mechanism was suggested in which the β-roll interactions were thought to play an important role in cyclic nucleotide binding affinity whereas interactions with the C-helix were described to be involved in efficiency of the allosteric transitions. In addition, using transition metal ion FRET it was demonstrated that agonist binding causes a stabilization of the C-helix in HCN2 [16, 28]. Additional evidence supporting an important dynamic role of the C-helix comes from X-ray crystal structures and NMR structures of the MlotiK1 CNBD. X-ray crystallography revealed 6 distinct conformations of the C-helix in MlotiK1, making it difficult to infer how the C-linker moves during channel gating [29]. NMR spectroscopy was employed to determine structures of the MlotiK1 CNBD in absence and presence of cAMP and demonstrated a movement of the A-, B- and C-helices with respect to the β-roll [30, 31]. Finally, a recent study employed double electron-electron resonance (DEER) spectroscopy and found motions up to 10 Å induced by cAMP binding in HCN2. The data further indicate a reorientation of several helices within the CNBD, including the C-helix [23].

Our results provide structural insight into the differences between the agonist and antagonist bound state of C-linker/CNBD from SthK. The structure of SthK-C_term_ in complex with cAMP, which is an agonist, reveals extensive ligand interactions with the β-roll and the C-helix from SthK-C_term_ which are similar to interactions that have been previously described for the cAMP bound HCN2 C-terminal structures. However, superposition of the cAMP bound HCN2 structure and the cAMP-bound SthK-C_term_ structure shows a prominent outward and upward shift at the aminoterminal end of the C-linker from the SthK-C_term_, which connects to the S6 segment of the transmembrane channel pore. This shift results in a greater distance (28.6Å) at the C-linker tetramer interface of SthK which mirrors the carboxyterminal residues of the S6 segment from the open gate structures of the K_v_1.2 channel [18] (29.7Å) or the MthK channel [18, 19] (30.3Å). This result suggests that the SthK structure may represent an activated or intermediate conformation, whereas previously published HCN structures have been suggested to represent an intermediate [9] or resting [32] conformation. Additionally, the structure of SthK-C_term_ in complex with cGMP, which is an antagonist, shows the SthK-C_term_ in a more closed conformation which is similar to the conformations which were previously shown for HCN2 C-terminal structures. Besides the conformational changes between SthK-C_term_ in complex with cGMP and cAMP, the structure of SthK-C_term_ in complex with cGMP also provides structural insights into antagonist binding in the C-linker/CNBD. In contrast to previously published HCN structures which showed the cGMP molecule bound in its syn-conformation [9, 12], the crystal structure of SthK-C_term_ shows a cGMP molecule bound in the anti-conformation. We observe that this conformation disables cGMP to establish the interactions with the C-helix as were found in the cAMP bound SthK-Cterm and the cAMP and cGMP bound HCN2 structures. Therefore, this differential orientation might explain why cGMP acts as an agonist in HCN2 channels but as an antagonist in the SthK channel. Moreover, the SthK-Cterm crystal structure suggests that binding of the cGMP molecule in the syn conformation might be prevented by the presence of the Leu-422 residue which enters the cGMP binding pocket and is equivalent to the Asp residue which has been described to play an important role in cGMP specificity [14, 26].

Together, the SthK crystal structures offer a framework for understanding conformational changes during ligand activation of cyclic nucleotide-modulated ion channels. We suggest a molecular mechanism of channel modulation in which binding of a cAMP molecule causes an outward swinging motion of the P-helix, together with the B- and C-helices of the CNBD. This movement is then transmitted through the D’-E’-F’-helices of the C-linker, which remain relatively constant. The outward movement of the C’-helix together with a pronounced bending of the A’-helix then creates a force to open the channel gate. Such a model for channel modulation is compatible with a previously suggested mechanism in which the CNBD imposes an inhibitory effect on the channel gate and ligand binding causes a relief of inhibition [33]. We suggest that the insight derived from the SthK-C_term_ crystal structures possibly extends to other cyclic nucleotide-modulated channels, including HCN and CNG channels. This work paves the way for crystallization efforts of the full-length SthK channel as well as further biochemical and spectroscopic studies, including NMR and DEER spectroscopy.

## Supporting Information

S1 Fig Electrophysiological properties of SthK: cAMP acts as an agonist, whereas cGMP behaves as an antagonist. (DOCX)

S2 Fig SEC-MALLS analysis of SthK-C_term_. (DOCX)

S3 Fig Comparison of the crystal packing for the two different crystal forms of SthK-C_term_ in complex with cAMP or cGMP. (PDF)
