# Supplementary material for: Structure of the SthK Carboxy-Terminal Region Reveals a Gating Mechanism for Cyclic Nucleotide-Modulated Ion Channels
Source: PLoS One. 2015 Jan 27;10(1):e0116369. doi: 10.1371/journal.pone.0116369 (PMC4308110; doi:10.1371/journal.pone.0116369)
Supplement: S1 Fig — (DOCX) [file pone.0116369.s001.docx]

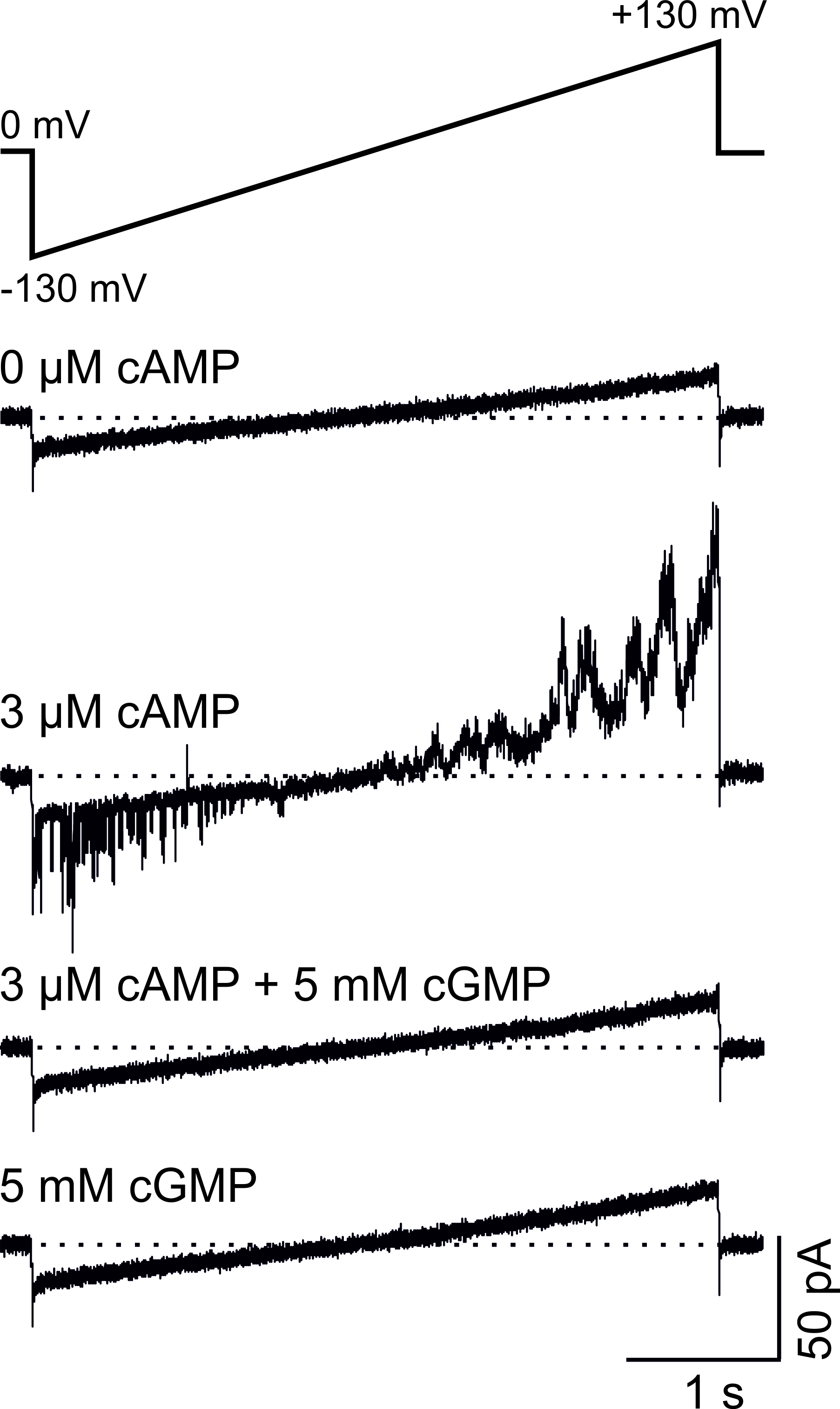


**Figure S1.** **Electrophysiological properties of SthK:** **cAMP acts as an agonist, whereas cGMP behaves as an antagonist.**

A voltage ramp ranging from -130 to +130 mV in 4.5 s was applied to an inside-out macropatch excised from a SthK-GFP–expressing *Xenopus laevis* oocyte. The representative traces show current responses after applying 3 μM cAMP, 3 μM cAMP plus 5 mM cGMP, and 5 mM cGMP, respectively. There was no current response as long as cGMP was applied. The absence of any current in the presence of 3 μM cAMP plus 5 mM cGMP clearly suggests a cGMP antagonism.
