# Supplementary material for: Structure of the SthK Carboxy-Terminal Region Reveals a Gating Mechanism for Cyclic Nucleotide-Modulated Ion Channels
Source: PLoS One. 2015 Jan 27;10(1):e0116369. doi: 10.1371/journal.pone.0116369 (PMC4308110; doi:10.1371/journal.pone.0116369)
Supplement: S2 Fig — (DOCX) [file pone.0116369.s002.docx]

| **a** | 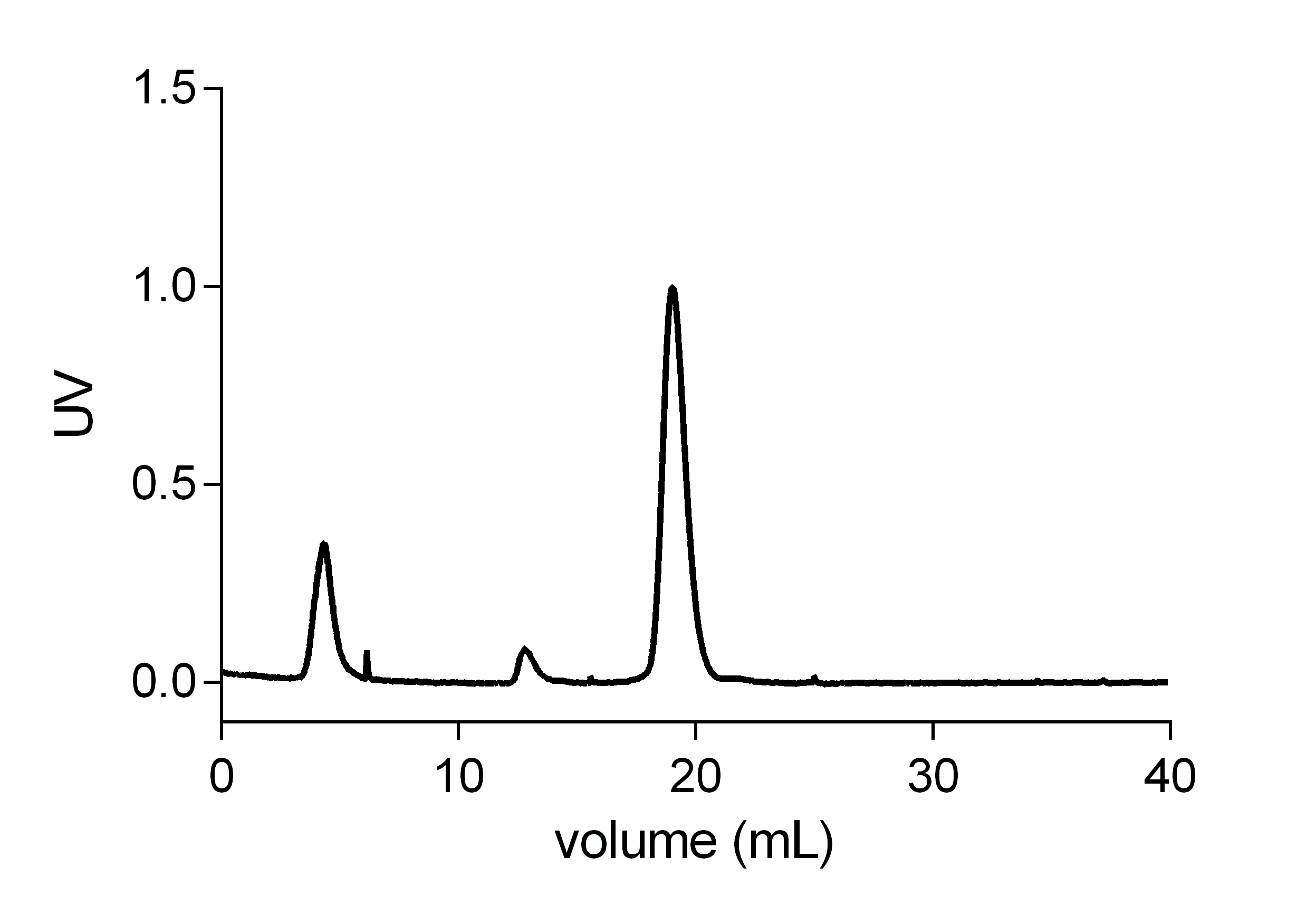 |
| --- | --- |
| **b** | 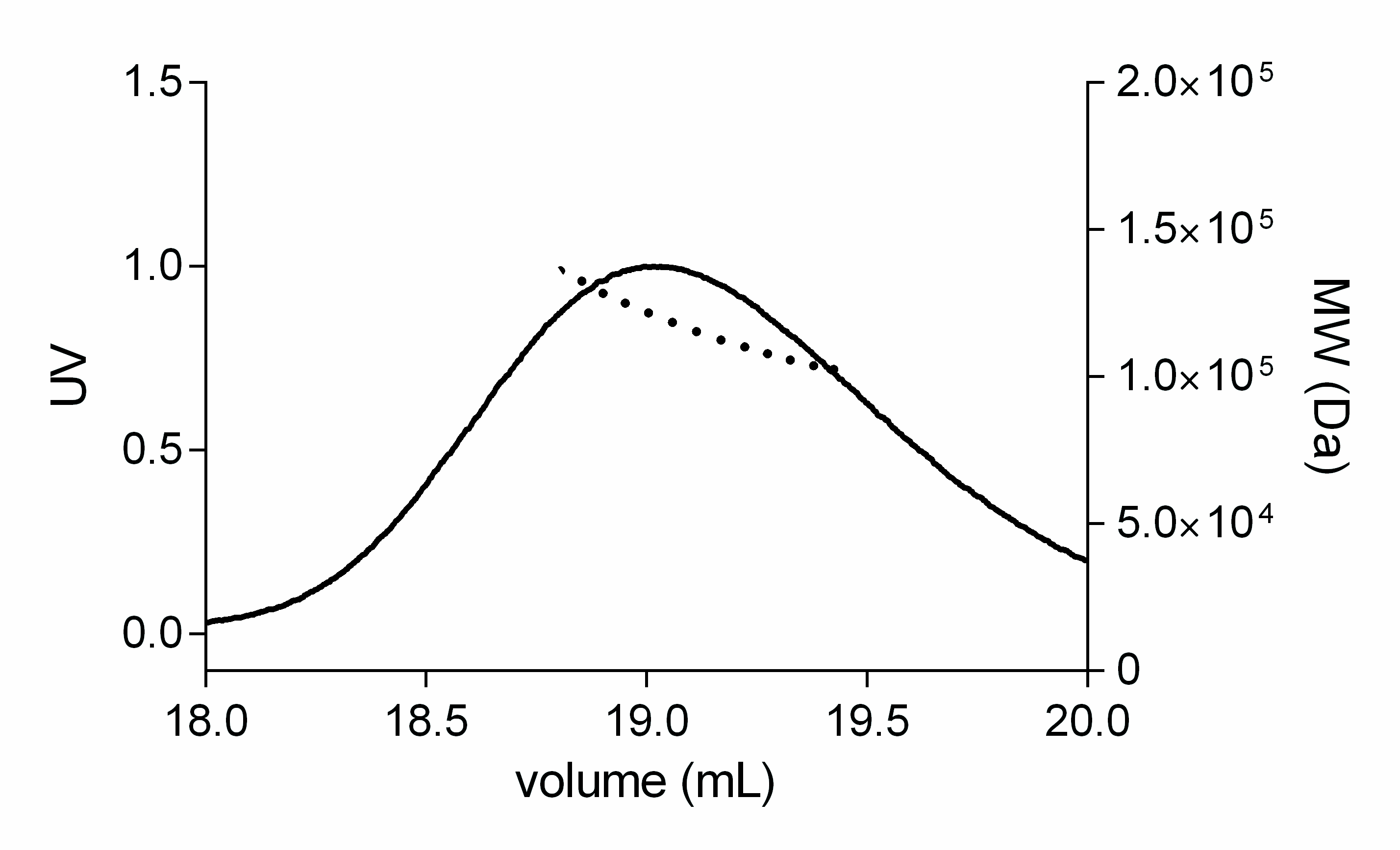 |

**Figure S2.** **SEC-MALLS analysis of SthK-C_term_.**

**a)** Size exclusion chromatography (SEC) profile of SthK-C_term_ on a Superdex 200 pre-equilibrated with buffer containing 30 mM HEPES pH 8.0, 500 mM NaCl, 1 mM DTT and 10% glycerol. **b)** Detailed zoom of the main peak eluting at ~19 ml. The MALLS-signal is plotted as a dotted line and indicates a size near 100 kDa, which matches to the theoretical size of tetrameric SthK-C_term_ (96.4 kDa).
