# Supplementary material for: Structure of the SthK Carboxy-Terminal Region Reveals a Gating Mechanism for Cyclic Nucleotide-Modulated Ion Channels
Source: PLoS One. 2015 Jan 27;10(1):e0116369. doi: 10.1371/journal.pone.0116369 (PMC4308110; doi:10.1371/journal.pone.0116369)
Supplement: S3 Fig — (PDF) [file pone.0116369.s003.pdf]

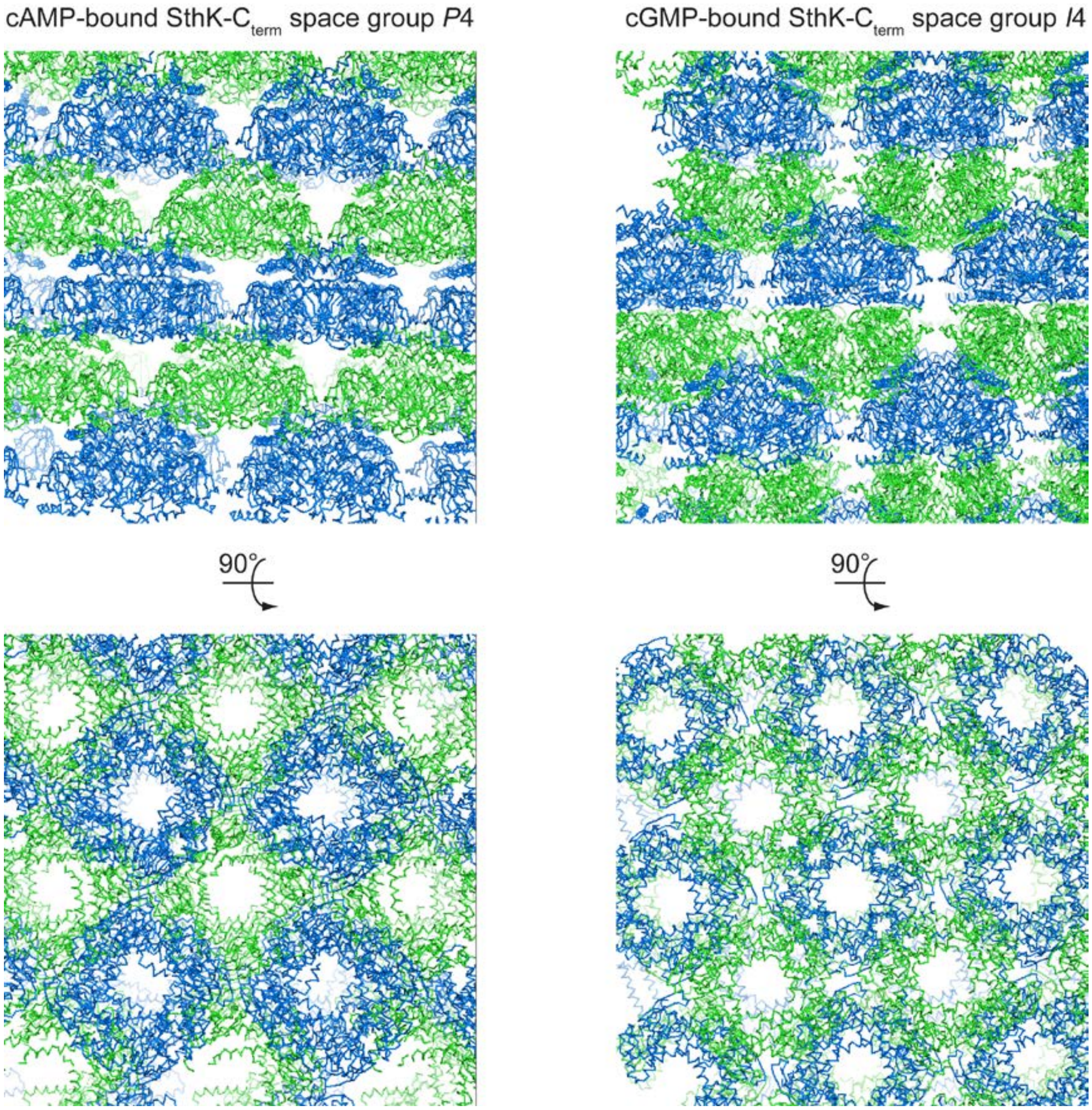

Figure S3. Comparison of the crystal packing for the two different crystal forms of SthK-C$_{term}$ in complex with cAMP or cGMP.

Chain A is colored in blue, chain B in green. In the cAMP-bound crystal form, different tetramers are stacked in a head-to-tail arrangement. In the cGMP-bound crystal form different tetramers are stacked in a tail-to-tail arrangement.
